# Synergistic Action of Montmorillonite with an Intumescent Formulation: The Impact of the Nature and the Strength of Acidic Sites on the Flame-Retardant Properties of Polypropylene Composites

**DOI:** 10.3390/polym12122781

**Published:** 2020-11-25

**Authors:** Raíssa Carvalho Martins, Michelle Jakeline Cunha Rezende, Marco Antonio Chaer Nascimento, Regina Sandra Veiga Nascimento, Simone Pereira da Silva Ribeiro

**Affiliations:** Instituto de Química, Universidade Federal do Rio de Janeiro, Cidade Universitária, CT, Bloco A, Rio de Janeiro, RJ 21941-909, Brazil; raissacarvalho1@gmail.com (R.C.M.); michelle@iq.ufrj.br (M.J.C.R.); chaer@iq.ufrj.br (M.A.C.N.); rsandra@iq.ufrj.br (R.S.V.N.)

**Keywords:** flame retardancy, intumescence, acidic montmorillonite, pyridine adsorption, polypropylene, synergistic agents

## Abstract

A raw montmorillonite (Mt) was submitted to different acidic activation times in order to investigate the influence of the strength and the nature (Brønsted and Lewis) of acidic sites on the synergistic action with an intumescent formulation (IF) composed of ammonium polyphosphate (APP) and pentaerythritol (PER) when incorporated into a polypropylene (PP) matrix. The acidity of the Mt samples was quantified by ammonia temperature-programmed desorption (TPD-NH3) and Fourier transform infrared spectroscopy (FTIR) with pyridine adsorption. The mineral clays were also characterized by X-ray fluorescence (XRF), X-ray diffraction (XRD), nitrogen adsorption analysis and particle size distribution. Thermogravimetric analysis (TGA), limit oxygen index (LOI) and UL-94 were performed to evaluate the flame-retardant properties and the thermal stability. The TGA results show that the final residue increased 2 to 3 fold in comparison to the values predicted theoretically. The flammability properties achieved a maximum for the system containing an excess of moderate-strength Brønsted sites relative to the Lewis ones, reaching 38% in the LOI test. This result suggests that the presence of these Brønsted acidic sites is important, as they take part in the esterification reaction between APP and PER which gives rise to the char formation. The FTIR-Pyr adsorption and flammability results indicate that both the nature and strength of the acidic sites influence the flame-retardant properties.

## 1. Introduction

Modern society depends upon polymers, mainly due to their versatility to build up new materials. However, polymers also have a significant disadvantage ascribed to their high flammability [1]. In order to reduce this drawback, several procedures have been developed, such as the use of reactive agents [2,3,4], flame-proof coatings [5,6,7] and flame-retardant additives. Among them, the addition of flame retardants to the polymer is the most commonly employed due to the low cost of the raw materials employed and of the process of incorporating them to the polymeric matrix [8]. Some of the currently most used flame retardant additives are mineral-based on metal hydroxides, e.g., Al(OH)_3_, halogenated, phosphorus-based, inorganic (e.g., borates) and the intumescent ones [8]. The latest ones present the great advantage of requiring lower loadings than the other minerals, which helps retaining the mechanical properties of the polymer [9]. Moreover, the intumescent additives do not release highly toxic gases, as the halogenated ones are much cheaper than the inorganic agents [8] and considered as a low environmental impact alternative [10].

**Figure 1:**
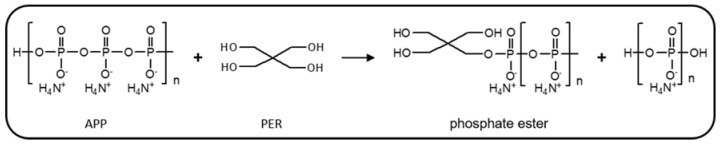
Reaction between ammonium polyphosphate (APP) and pentaerythritol (PER). Adapted from Camino et al. [14].

An intumescent system consists mainly of a blowing agent, an acid source and a carbonific agent [11]. Bourbigot et al. [12] and Camino et al. [13] have extensively studied the intumescent system composed of ammonium polyphosphate (APP), which works as both the blowing agent and the acid source, and pentaerythritol (PER), which plays the role of the carbonific agent. Bourbigot et al. [12] proposed that as the temperature increases, degradation of APP takes place, forming acidic species such as phosphoric acid and pyrophosphates. These species react with PER, a polyhydroxylated compound, forming phosphate esters at a temperature lower than 280 °C. On the other hand, Camino et al. [14] suggested the direct reaction between APP and PER, without the previous degradation of the former, as shown in Figure 1, to form phosphate esters. According to Delobel et al. [15], in the temperature range of 280 °C < T < 350 °C the phosphorus esters decompose generating unsaturated compounds which, through Diels–Alder reactions, produce aromatic compounds that are the precursors to the formation of a carbonaceous layer, the char. The blowing agent decomposes, generating gaseous products that will cause the char to swell, forming an intumescent protective layer which acts as a physical shield, avoiding the interchange of oxygen, heat and fuel between the material surface and the outside, thus breaking down the fire feedback cycle. However, above 430 °C the layer becomes stiffer [12], generating cracks and allowing the diffusion of combustible materials to the flame.

Montmorillonite (Mt) has been widely investigated as a mineral filler in intumescent flame retardants due to its power in reducing polymer flammability, even when added in small amounts to the polymeric composite [16,17,18]. Among Mt’s advantages should be highlighted not only its ability to retard heat transfer between the environment and the polymeric material, but also its ability to lower the supply of volatile products released to the gas phase [19]. Fina et al. [20] concluded that the way Mt migrates to the polypropylene surface influences the diffusion of oxygen and, consequently, the composite’s ignition time. Furthermore, due to their low cost, easy access and low environmental impact, clay minerals are largely investigated as solid catalysts [21], especially Mt, whose surface acidity can be modified by mineral acid treatment, enhancing their catalytic and adsorption properties for use in organic reactions [22], besides presenting swelling and cation exchange capacity [21]. Zanetti et al. [23] studied the thermal behavior of layered silicate ethylene-vinyl acetate (EVA) nanocomposites and observed the catalytic effect promoted by the strongly acid sites created due to the thermal decomposition of the silicate modifier when in contact with the polymer. Also, Tang et al. [24] investigated intumescent PP/Mt nanocomposites and observed not only the synergistic effect between Mt and the intumescent formulation, but also the catalytic effect addressed to the decomposition of the amine Mt modifier, which would produce strong acid catalytic sites, contributing to the char formation through dehydrogenation-crosslinking reactions.

Nevertheless, to the best of our knowledge, no work has investigated in depth the influence of the nature and the strength of the acidic sites of non organomodified solid catalysts on the flame-retarding properties of intumescent composites. Recently, our group published two pioneer studies about the effect of the acidity of zeolites on the synergistic action with a polypropylene (PP) composite containing APP and PER as the intumescent formulation. Bernardes et al. [25] showed that increasing the concentration and accessibility of the acidic sites of H-ZSM-5 zeolites improves the flame-retardant properties of the composites. Ribeiro et al. [26] showed that, for faujasite Y zeolites, moderate strength acidic sites are responsible for a better performance in the flammability tests, while stronger ones impair the flame-retarding properties.

The present work addresses the effect of the strength and the nature (Brønsted and Lewis) of the acidic sites of raw and acidic-activated Mt on the synergistic action with an intumescent formulation composed by APP and PER in a PP matrix.

## 2. Materials and Methods

### 2.1. Raw Materials

The raw Mt used is from Boa Vista district, Paraíba, Brazil. Sulphuric acid (95–99%) was purchased from Vetec (Sao Paulo, Brazil). The commercial clay mineral K10 was supplied by FLUKA (Zurich, Switzerland). Ammonium polyphosphate (APP), (trade name Exolit 422) was purchased from Clariant (Muttenz, Switzerland), pentaerythritol (PER) from Sigma Aldrich (San Luis, MO, USA), and polypropylene (PP) (code 448R) was supplied by Braskem (Sao Paulo, Brazil).

### 2.2. Montmorillonite’s Acidic Activation

The raw sodium clay mineral was completely crushed and passed through a 0.250 mm sieve opening. The sample was divided into four parts. The first part was kept as supplied, while the other ones were submitted to an acidic activation with 4 mol L^−1^ H_2_SO_4_ solution, for 2, 5 and 20 h, at a concentration of 10% m/v mineral clay, as described by Rezende et al. [27]. The clay mineral sample in the first group was labelled as ANa, and the acidified ones were labelled as AH2, AH5 and AH20, respectively.

### 2.3. Montmorillonite´s Chemical Composition: X-ray Fluorescence (XRF)

In order to determine the chemical composition, 0.5 g of each clay mineral sample dried at 100 °C was mixed to 5 g of flux using a borate mixture (1:10 dilution) and submitted to melting at 1050 °C in an Eagon Machine. The resulting samples were analyzed on an X-ray fluorescence spectrometer (WDS-2), model AxiosMax (Panalytical B.V., Almelo, The Netherlands).

### 2.4. Montmorillonite’s X-ray Diffraction (XRD)

The samples’ X-ray diffractograms were collected on a Bruker-AXS D8 Advance Eco (Bruker AXS GmbH, Karlsruhe, Germany) with Cu k radiation, operating at the following conditions: generator at 40 kV and 25 mA; goniometer velocity of 0.02° s^−1^ and 2θ range from 1 to 40°. The qualitative interpretation was performed using the Bruker AXS Diffrac. Plus software. The interlayer spaces (d) were calculated according to Bragg’s law.

### 2.5. Montmorillonite’s Textural Properties

The textural properties (specific surface area, pore diameter and volume) were calculated through low-temperature nitrogen adsorption-desorption isotherms using a Quantachrome Nova 1200 adsorption analyser (Quantachrome Instruments, Boynton Beach, FL, USA). The Mt samples were degassed in vacuum for 24 h at 120 °C prior to nitrogen adsorption. The specific surface area was calculated according to the standard Brunauer–Emmett–Teller (BET). The pore diameter and volume were determined from desorption isotherms using the following calculation model: N_2_ at 77 K on silica, cylindrical pore, non-linear density function theory (NLDFT) equilibrium model.

### 2.6. Montmorillonite’s Particle Size

The particle size distribution was determined by laser diffraction, through the Mastersizer 2000 analyser (Malvern Panalytical, Almelo, The Netherlands). The parameters used in the calculations were the normal sensitivity and the equivalent spherical particle. The Fraunhofer theory was used for calculating the granulometric distribution curves.

### 2.7. Montmorillonite’s Acidity Quantification

To determine the total acidity, i.e., the total number of the clay minerals’ acidic sites, the ANa, AH2, AH5, AH20 and K10 samples were analyzed by temperature programmed desorption of ammonia (TPD-NH_3_), employing a Zeton-Altamira, model AMI-90 (Zeton Altamira, Pittsburgh, PA, USA), with a thermal conductivity detector. The samples were pre-treated at 250 °C using a heating rate of 10 °C min^−1^ under helium flow at 30 mL min^−1^. Next, the samples were cooled to 175 °C and subjected to chemisorption of NH_3_ by pulses with fixed volumes of the gas. The desorption process was carried out between 175 °C and 500 °C, at 10 °C min^−1^, with isotherm at 500 °C, for 120 min.

Fourier transform infrared (FTIR) spectroscopy with pyridine adsorption was used to distinguish Lewis and Brønsted acidic sites. IR experiments were conducted using a FT Spectrometer, Perkin Elmer model Spectrum 100 (PerkinElmer Life and Analytical Sciences, Shelton, CT, USA), equipped with a DTGS detector, self-supported wafers (with 1.6 cm diameter and mass varying from 16 to 36 mg), and a Pyrex cell with CaF_2_ windows. The covered spectral range was from 4000 to 1000 cm^−1^ with a 4 cm^−1^ resolution. The samples were pre-treated at 150 °C for 1 h in vacuum up to 1.35 × 10^−8^ kgf cm^−2^. After cooling to room temperature, the infrared spectrum was recorded and used as background for the adsorption experiments. These studies were conducted by exposing the pre-treated wafers to 1.35 × 10^−2^ kgf cm^−2^ of pyridine at 0 °C for 30 min, followed by evacuation up to 1.35 × 10^−8^ kgf cm^−2^ at the same temperature. The difference spectra, obtained by subtracting the spectrum of the dehydrated sample from those after pyridine adsorption, were used to obtain the Brønsted and Lewis sites concentration by integration of the characteristic regions. Desorption was performed at 250 °C, 350 °C and 450 °C for 30 min at each temperature.

### 2.8. Processing of the Composites

PP, APP, PER and the clay minerals (ANa, AH2, AH5, AH20 and K10) were used to produce the polymeric composites. Two composite groups were studied, one containing the intumescent formulation (IF), i.e., with APP+PER, and the other without it. For the systems with the IF, a mass/mass proportion of 67% PP:30% IF:3% clay mineral was used, while for systems without the IF, the proportion was 97% PP:3% clay mineral. In the first system, a 3APP:1PER mass ratio was used.

The polymeric materials were processed in a Thermo Scientific Haake Rheomex double screw extruder model PTW16 (Thermo Fisher Scientific Inc., Waltham, MA, USA). The extruder has six heating zones that were kept at the following temperatures: T_zone 1_ = 90 °C, T_zone 2_ = 135 °C, T_zone 3_ = 170 °C, T_zone 4_ = 180 °C, T_zone 5_ = 190 °C, T_zone 6_ = 190 °C. The materials were fed into zone 1, with 13% feeding and a thread rotation of 300 rpm. After extrusion, the materials were pelletized in a Brabender Nord instrument (Brabender GmbH & Co. KG, Duisburg, Germany) and the pellets were pressed using a Carver Laboratory Press, model C, at 220 °C, under 6000 lbf for the first 4 min and 12,000 lbf for the remaining 6 min.

### 2.9. Polymer Composites Scanning Electron Microscopy (SEM)

In order to observe the changes in the aspect of the composites’ surfaces, samples with 100 × 7 × 3 mm dimension, with and without intumescent formulation, were burned in a 2 cm long blue flame of butane for 10 s and analyzed on a Phenom ProX scanning electronic microscope (Thermo Fisher Scientific Inc., Waltham, MA, USA), operating at 10 kV and with a secondary electrons detector. Moreover, the surface of the composite samples with and without the IF were also analyzed by SEM before being burnt.

### 2.10. Polymer Composites Thermogravimetric Analysis (TGA)

The composites obtained were immersed in liquid nitrogen and then milled in order to be submitted to TG measurements. All the components, individually and in combinations, were analyzed using a TA Instruments Simultaneous DSC-TA, model QD600SDT (TA Instruments: New Castle, DE, USA), and the following operation conditions: 10 mg sample, alumina microbalance pan, 40 °C min^−1^ heating rate, 50 cm^3^ min^−1^ synthetic air flow. The experimental and theoretical thermogravimetric analysis (TGA) and dTG curves were presented in order to verify the existence of any synergism among the components of the composites. The theoretical curves take into consideration the individual contribution of each additive in its respective concentration to the composite’s TGA curve, according to Table 1 [11].

### 2.11. Polymer Composites Flammability Tests

#### 2.11.1. Underwriters Laboratory UL-94

An Underwriters Laboratory’s UL-94 test (vertical burning test; ASTM D 380) was performed to measure the ease of ignitibility of the materials according to their burning characteristics, as well as the time spent to extinguish the combustion after the heat source removal. Five specimens (100 mm × 13 mm × 3 mm) for each sample were used for this test. The specimen was held vertically for 10 s with its bottom in contact with a blue, 20 mm-long flame originating from the methane burn through a Bunsen burner, producing (or not) an after-flame (flame which persists after the ignition source has been removed). Once this after-flame was extinguished, this protocol was repeated a second time and the combustion times after the first and the second methane flame approximation (t1 and t2, respectively) were then recorded. Moreover, the incandescence time (t3) after the extinction of the after-flame was recorded. Also, other phenomena are taken into account, such as the occurrence of dripping (ignition of the cotton batting placed 300 mm beneath the specimen) and burnt extension of the specimen (combustion up to the holding clamp). The best rating was V0, i.e., when the material rapidly extinguished the combustion flame, besides presenting very little afterglow without producing burning drips. The worst classification was V2, meaning a poor flame-retarding property of the material. The criteria used for the material classification is shown in Table 2 [28].

#### 2.11.2. Limit Oxygen Index (LOI)

The limit oxygen index (LOI; ISO 4589-2) determines the minimum oxygen content in a mixture of oxygen and nitrogen required for maintaining the burning process. Through this test it is possible to assess how easily the polymeric materials ignite by applying a flame of standard length over an upright positioned specimen in a controlled atmosphere. This test was performed in a fire-testing technology (FTT) apparatus (Fire Testing Technology, East Grinstead, UK) using 100 mm × 7 mm × 3 mm specimens of the neat polymer and of the produced composites. A propane flame was brought close to the top of the sample each 5 s during 30 s and its fire behavior observed. After ignition at a certain concentration of oxygen, the sample should extinguish the flame in less than 3 min without having half of its length consumed. The test was repeated for increasing concentrations of oxygen until those criteria were no longer reached. The higher the %O_2_, the better the flame-retarding properties, i.e., a greater amount of oxygen would be necessary to maintain the combustion within a certain environment, keeping in mind that the average concentration of oxygen in the atmosphere is 21%.

## 3. Results and Discussion

### 3.1. Montmorillonite’s Chemical Composition: X-ray Fluorescence (XRF)

The chemical composition of the samples ANa, AH2, AH5, AH20 and K10 are presented in Table 3. As the time of acidic activation increases, one observes a gradual lixiviation of magnesium, aluminum and iron from the Mt octahedral sheet. On the other hand, the exchange of sodium from the interlamellar space by acidic protons proved to be efficient, its percentage dropping abruptly from 0.48% m/m (Na_2_O) to less than 0.1%.

### 3.2. Montmorillonite’s X-ray Diffraction (XRD)

**Figure 2:**
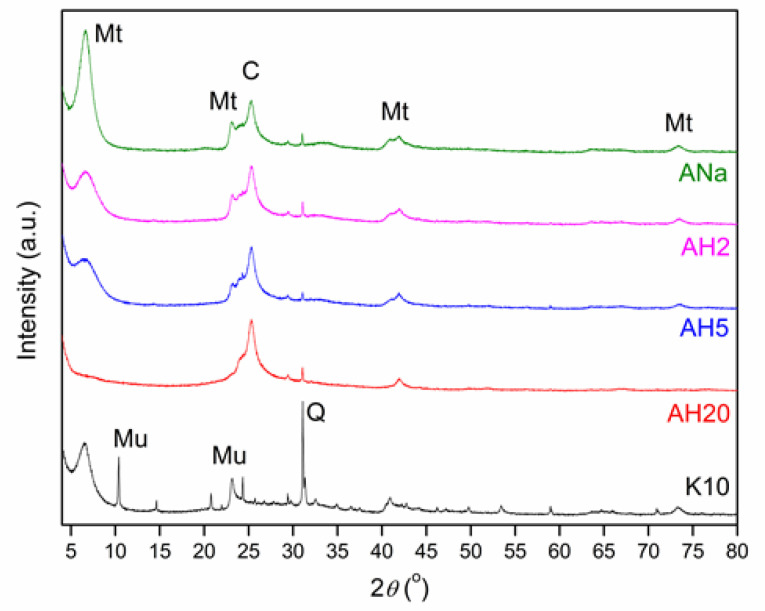
X-ray diffractograms for the montmorillonite samples. Mt stands for montmorillonite: (Na,Ca)0.3(Al,Mg)2Si_4_O_10_(OH)_2_.nH_2_O; C for cristobalite: SiO_2_; Mu for muscovite: KAl_2_(Si_3_Al)O_10_(OH)_2_ and Q for quartz: SiO_2_.

Figure 2 shows a comparison between the X-ray diffractograms of the Mt samples. The main Mt reflection occurs at 2θ = 6.63° (interlayer space of 13.33 Å) for the ANa sample and at 2θ = 6.51° (interlayer space distance of 13.57 Å) for the K10 sample. The ANa interlayer space remains the same for the AH2 and AH5 samples with a gradual loss of Mt organization as the acidic treatment takes longer. For the AH20 sample, that main reflection is not observed any more, indicating that the Mt structure was destroyed with further acidic treatment (20 h).

### 3.3. Montmorillonite’s Textural Properties

Table 4 shows a comparison of the textural properties of the clay’s samples. Along the acidic activation, the specific surface area increases from 104 m^2^ g^−1^ in ANa to 145 m^2^ g^−1^ in AH2 sample, but it drops gradually for AH5 (137 m^2^ g^−1^) and AH20 (87 m^2^ g^−1^). K10 presents the highest specific surface area (178 m^2^ g^−1^). The average pore diameter remains constant (51–53 Å) for ANa, AH2, AH5 and K10, rising significantly for AH20 (139 Å). The pore volume is kept constant (around 0.2 cm^3^ g^−1^) for all the samples.

Steudel et al. [29] observed that the maximum value for the specific surface area for different bent samples was obtained in different times after acidic activation with 5 mol L^−1^ H_2_SO_4_ solution for 1.5, 5, 20, 72 and 96 h at 80 °C. They also observed that a longer acid attack decreased the specific surface area while keeping the pore volume constant. They suggested that the initial increase in the specific surface area is due to the splitting of smectite particles (either by distortions caused by the unbalanced charge distribution between octahedral and tetrahedral sheets, or by the exchange of the interlayer cations and the protonation of Si–O groups of the tetrahedral sheet at pH below 2). The prolonged acidic treatment would then dissolve small particles (the octahedral sheets), releasing Al, Fe and Mg and decreasing the specific surface area. These results corroborate the XRD and XRF results showing that, despite the acidic activation, there is still an organization of the Mt crystalline phase for samples AH2 and AH5, which is completely lost in the AH20 sample.

### 3.4. Montmorillonite’s Particle Size

**Figure 3:**
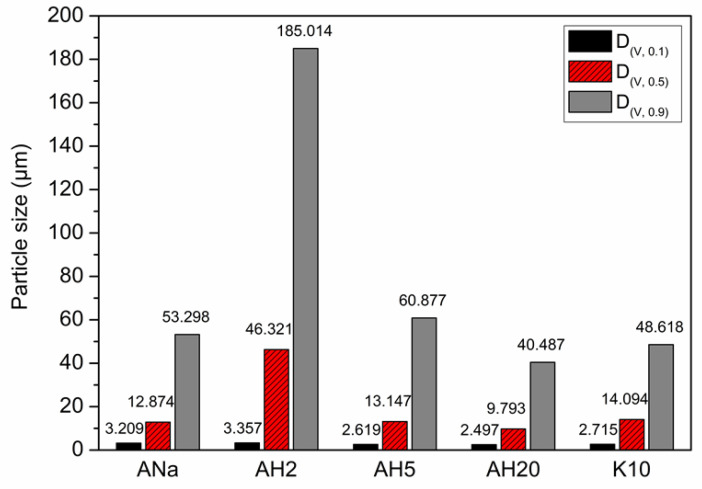
Particle size analysis of the clay minerals through the parameters D_(V, 0.5)_, D_(V, 0.1)_ and D_(V, 0.9)_.

Figure 3 presents the results of the particle size analysis of the clay minerals, through the parameters D(V, 0.5), D(V, 0.1) and D(V, 0.9). For ANa, which did not undergo acidic activation, 10% of the particles possess diameter under or equal to 3.209 µm (D(V, 0.1)), whereas 50% of the particles are under 12.874 µm (D(V, 0.5)) and 90% are under 53.298 µm (D(V, 0.9)). The acidic activation for 2 h leads to an increase in the particle size dispersion, as the parameter D(V, 0.9) increased to 185.014 µm, indicating the agglomeration of the particles. With further acidic treatment (5 h), the particle size dispersion tends to become more homogeneous again, dropping to values close to that of the raw clay mineral ANa. However, a longer acidic treatment (20 h, for AH20) seems to decrease even more the particle size dispersion and the D(V, 0.9), probably due to dissolving of the small particles (the octahedral sheets), as observed by Stuedel et al. [29]. K10 presents values for D(V, 0.5), D(V, 0.1) and D(V, 0.9) close to those for ANa.

### 3.5. Montmorillonites Total Acidity: Temperature-Programmed Desorption (TPD)-NH_3_

**Figure 4:**
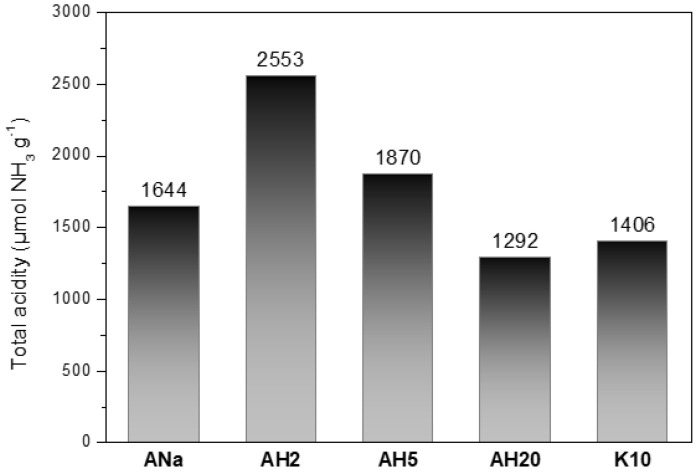
Total number of acidic sites for the samples ANa, AH2, AH5, AH20 and K10 determined by temperature-programmed desorption (TPD)-NH_3_.

The total number of acidic sites for the samples ANa, AH2, AH5, AH20 and K10 was determined in µmol of desorbed NH_3_ per gram of the sample. Figure 4 shows that the clay mineral AH2 presents the greatest concentration of acidic sites and AH20 the lowest one. Between these two, in decreasing order of concentration, one finds: AH5, ANa and K10. Thus, clay minerals AH2 and AH5 have a higher concentration of acidic sites than ANa, showing the effectiveness of the acidic treatment on the ANa. On the other hand, the low value of acidity found for AH20 can be attributed to the lixiviation of the cations present in the octahedral sheet and the consequent loss of structure, as evidenced by the XRF and XRD analyses.

### 3.6. Char’s Morphology: Scanning Electron Microscopy (SEM)

**Figure 5:**
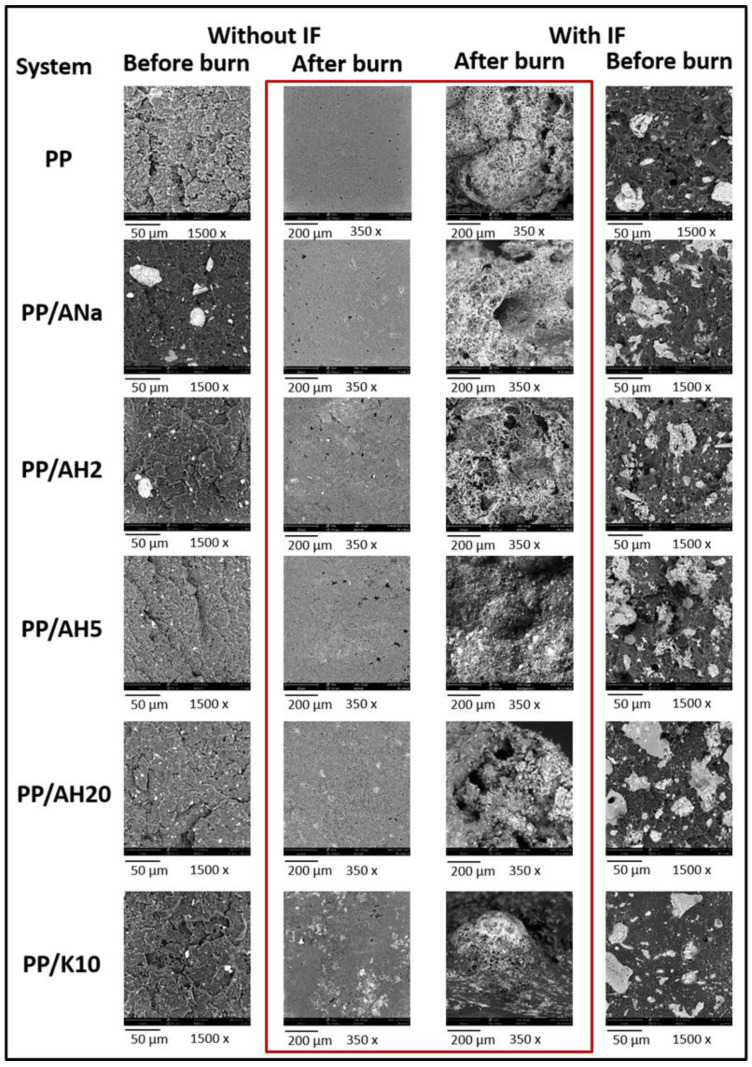
Scanning electron microscopy (SEM) images of surfaces of pure PP and polymeric clay mineral composites with and without intumescent formulation, before and after being burnt.

The surfaces of the composite samples with and without the IF were analyzed by SEM before and after being burnt (Figure 5).

Before they burn, the samples without IF showed some agglomerates of clay mineral in the polymeric matrix. It seems that the particles of AH5 and AH20 were better dispersed than those of ANa and AH2, as the first two passed through a more severe acidic activation and, consequently, were more exfoliated. For the non-burnt samples containing IF, agglomerates of APP, PER and clay minerals are observed, but there is a significant change in the dispersion of particles among the composites.

The burnt samples not containing IF exhibit a flat and smooth surface, while the burnt sample surfaces with IF were very rough and porous, with a multilayer aspect consisting of tiny gas bubbles trapped within their respective structures, as described by Bourbigot et al. [12]. Char formation was thus responsible for the residue boost after burning, as it works as a protective layer between the inner polymeric matrix and the outside environment, avoiding heat, oxygen and volatiles interchange among them. In the sample PP/IF/AH20 the presence of large holes was remarkable, indicating the formation of a poor-quality char, as the holes work as channels enabling the transportation of heat and fuel between the inner and outer sides of the composites. This particular morphology can indicate a poor performance of the flame-retardant properties. Thus, the quality of the char could not be directly related to the particle distribution in the PP matrix. On the other hand, the absence of roughness and porosity as in the non-intumescent composites may be correlated to the lack of flame-retarding properties.

### 3.7. Thermal Behavior and Stability: TGA

**Figure 6:**
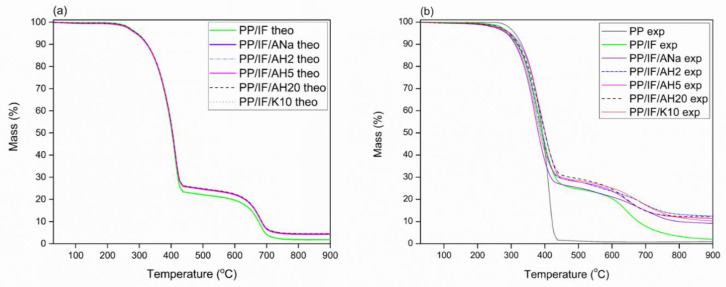
Thermogravimetric analysis: (**a**) theoretical TGA curves; (**b**) experimental TGA curves of neat PP, PP/IF and PP/IF/clay minerals.

Figure 6a,b present the theoretical and experimental TGA curves for the systems studied, respectively. The theoretical ones consider the contribution of each additive in its respective concentration to the composite TGA curve. In the absence of synergism between the components, the experimental curve should be identical to that calculated theoretically, i.e., just a sum of the individual contributions. On the other hand, the experimental and theoretical curves should show a different behaviour in the presence of synergism, in this case evidencing a better thermal stability, as the combined effect of the components is greater than the simple sum of their individual contributions. Figure 6a shows that the predicted theoretical behavior for all the PP/IF/clay minerals systems. They are very similar, with the curves well overlapped, and their profiles are identical to that of the PP/IF one. Only a slightly higher thermal stability is expected between 450 °C and 900 °C with a small increase of the final residue. However, when the experimental TGA curves for pure PP, PP/IF and PP/IF/clay minerals systems are plotted (Figure 6b), quite different behavior is observed.

The comparison between the PP and PP/IF curves shows that the neat polypropylene thermal degradation occurs in a single step in the range 300–450 °C, whereas the PP/IF system shows more than one degradation step. After the addition of clay mineral, a shift to higher degradation temperatures is observed in the 600–700 °C range when compared to the PP/IF composite, but the profiles of the PP/IF/clay minerals did not change relative to that of the PP/IF one, independent of the acidity. In the samples containing the intumescent formulation, the first step occurs between 250 and 450 °C, when the curves tend to level up until 600 °C, followed by a second smaller degradation step until 900 °C. The thermal stability between 450 °C and 600 °C is associated with the charred structure solidification and increases significantly when the clay minerals were added to the system, with a larger amount of remaining residue at 900 °C. As shown in Table 5, the AH2 system presents the highest experimental residue, followed by the systems with AH20, AH5, K10, ANa and the system without clay mineral in the decreasing order.

The addition of clay mineral clearly promotes an increase of the final residue, even if present in 3% m/m ratio in the composites, improving the char thermal stability, a factor that contributes to enhancing the fire-retardant properties, and corroborating previous results [16]. The fact that AH2 and AH20 present virtually the same amount of final residue but with a very different concentration of acidic sites (2553 and 1292 µmol NH_3_ g^−1^, respectively) does not allow a straightforward correlation between the concentration of acidic sites and the increase of the thermal stability. Fina et al. [20] observed that the increase in dispersion of exfoliated Cloisite 20A in a polypropylene matrix contributed to the thermal stability of the composite, as the migration of clay minerals sheets towards the composite surface during heating creates an insulating clay mineral barrier. This phenomenon is a possible explanation for the high thermal stability achieved by PP/IF/AH20, which is highly exfoliated after the severe acidic activation, as discussed in Section 3.1.

**Figure 7:**
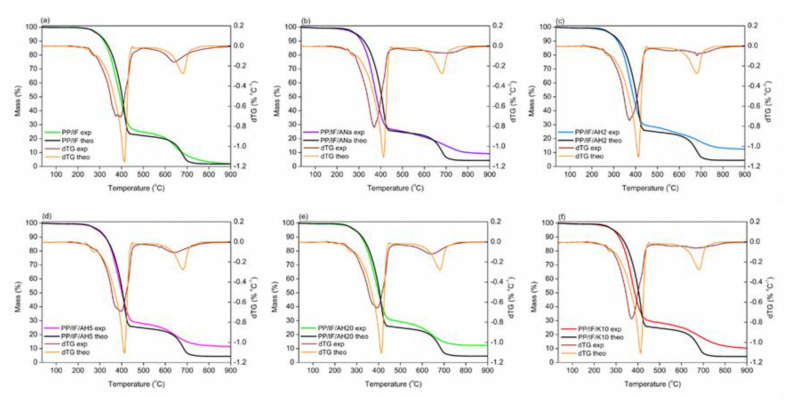
Comparison between the experimental and theoretical TGA and dTG curves for PP/IF and PP/IF/clay minerals systems. Left Y axis: Mass (%). Right Y axis: dTG (% °C^−1^): (**a**) composite without clay mineral; (**b**) composite with Ana; (**c**) composite with AH2; (**d**) composite with AH5; (**e**) composite with AH20; (**f**) composite with K10.

In Figure 7a–f, the experimental and theoretical TGA and dTG curves for each system were individually compared in order to evaluate the synergism among the components of the composites. Both experimental and theoretical data are transcribed in Table 5. From the results in Table 5 it is clear that, for all the systems in the 300–400 °C range, the experimental temperature of the first most intense dTG peak (T_peak1_) has shifted to a lower value relative to the respective theoretical one. Moreover, the experimental T_onset_ are also lower in the experimental curves than in the theoretical ones.

The synergistic effect between the IF and the clay minerals becomes evident when the final residue for both theoretical and experimental TGA curves are compared, as presented in Table 5. The experimental and theoretical residues for the PP/IF system are identical. With the addition of the clay minerals, the experimental residues are two to three times higher than those predicted theoretically. Moreover, the addition of the clay minerals increases the overall thermal stability of the system, avoiding the complete degradation of the intumescent layer, as evidenced by the smoothness of the second degradation step (around 700 °C). Moreover, after the second degradation step, a higher % of residue was obtained for the PP/IF/clay mineral systems than was theoretically expected, and even higher than the experimentally observed for the PP/IF system.

### 3.8. Flammability Tests

#### 3.8.1. UL-94

All the samples containing IF, even without the addition of clay mineral, achieved the best UL-94 classification, V0, meaning that, for each group of 5 specimens, the after-flame was quickly extinguished (in less than 10 s), the incandescence lasted less than 30 s and that neither combustion up to the holding clamp nor burning dripping were observed. The addition of clay mineral did not change the maximum UL-94 classification reached by the PP/IF sample. Tests for neat PP and for composites containing only PP and clay mineral (ANa, AH2, AH5, AH20 and K10, without IF) were also performed and none of them was classified in the UL-94 test, making it clear that the addition of the intumescent formulation is crucial in order to achieve flame-retarding properties.

#### 3.8.2. Limit Oxygen Index (LOI)

The LOI tests for the PP composites, containing or not the intumescent formulation, were performed and the results are shown in Table 6. The higher % LOI better the flame-retarding properties, i.e., the greater the amount of oxygen necessary to maintain the combustion within a certain environment, keeping in mind that the average concentration of oxygen in the atmosphere is 21%. For the samples without IF, there was no significant improvement in the flame-retarding properties, as the addition of clay mineral increased the LOI value of neat PP (17% O_2_) by only one LOI unit (18% O_2_). In contrast, the sole addition of IF to PP raised the LOI value to 30% O_2_, corresponding to an increase of 13 LOI units. Moreover, the addition of clay minerals ANa, AH2, AH5 and K10 proved to enhance even more the flammability properties, reaching LOI values of 33 (for K10), 35 (for ANa and AH2) and 38% O_2_ (for AH5). Although the clay mineral by itself in the PP matrix does not cause any significant effect, there is considerable improvement when the clay mineral/IF combination is used. Once again, the synergism between the clay minerals and the intumescent formulation is evident. Curiously, this improvement was not observed for PP/IF/AH20 system, which presented a LOI value of 27% O_2_, lower than that obtained for the PP/IF/ sample (30% O_2_), showing an antagonist interaction with the APP-PER formulation for this test. It is important to recall that the SEM images for PP/IF/ AH20 sample showed a poor formed char, with large holes on its surface (Figure 5).

Despite the large difference between the concentration of acidic sites in the AH2 (2553 µmol NH_3_ g^−1^) and ANa (1644 µmol NH_3_ g^−1^) samples, their respective intumescent composites achieved the same LOI result (35%). Furthermore, the intumescent composite containing AH5, whose concentration of acidic sites is between those values (1870 µmol NH_3_ g^−1^), presented the highest LOI value (38%). An attempt at correlating LOI and clay mineral TPD-NH_3_ results failed, up to this point, to explain the LOI index behaviour of the PP/IF/clay mineral samples. Also, it was not possible to establish a direct correlation between either the changes in the textural properties or the gradual loss of the Mt organization along the acidic treatment with the different performances achieved by the composites in the LOI test. Furthermore, in comparison to the raw mineral clay ANa, the particle size distribution changes abruptly for AH2 and varies slightly for AH20, but it does not show a significant variation among ANa, AH5 and K10. Therefore, the particle size distribution cannot justify the different flame-retarding performances achieved by the composites. Thus, for a deeper understanding about the nature of the acidity sites of the clay minerals it was necessary to submit the samples to FTIR spectroscopy with pyridine adsorption.

#### 3.8.3. Montmorillonite’s Brønsted and Lewis Acidity: Fourier Transform Infrared Spectroscopy with Pyridine Adsorption (FTIR-Pyr)

Pyridine is widely employed as a reliable probe molecule in order to investigate both quantitatively and qualitatively the acidic nature of Brønsted and Lewis sites in solid catalysts such as zeolites and clay minerals [21,30,31,32,33,34,35,36,37]. As thoroughly explained by Busca et al. [38] for ionic oxides, surface exposed cations which are coordinatively unsaturated give rise to Lewis acid sites, while Brønsted acidic sites arise from hydroxyl groups produced by the dissociative water adsorption on the surface of metal oxides. The difference in the interaction with those sites is responsible for vibrations in the region between 1700–1400 cm^−1^ due to the pyridine ring deformation, enabling both the Brønsted and Lewis surface sites to be detected and characterized [39]. The band at 1545 cm^−1^ is attributed to pyridine adsorbed on Brønsted acidic sites (Bpy), while the IR band at 1450–1455 cm^−1^ accounts for pyridine coordinated to Lewis acidic sites (Lpy). A strong band at 1490 cm^−1^ is related to pyridine associated with all acid sites, including hydrogen bonding between the pyridine nitrogen atom and –OH groups present on the clay mineral surface, HPy sites [22]. Among them, the bands at 1545 cm^−1^ and 1455 cm^−1^ are reliable for quantifying Brønsted and Lewis acidic sites, respectively, as they are those best resolved in the analyzed spectral range [30]. Moreover, the FTIR-Pyr technique also provides useful information on the strength of the acidic sites when desorption is carried out at different temperatures. The higher the temperature in which pyridine is retained, the stronger the acidic site is considered to be, as more energy is necessary to desorb pyridine from that particular site.

**Figure 8:**
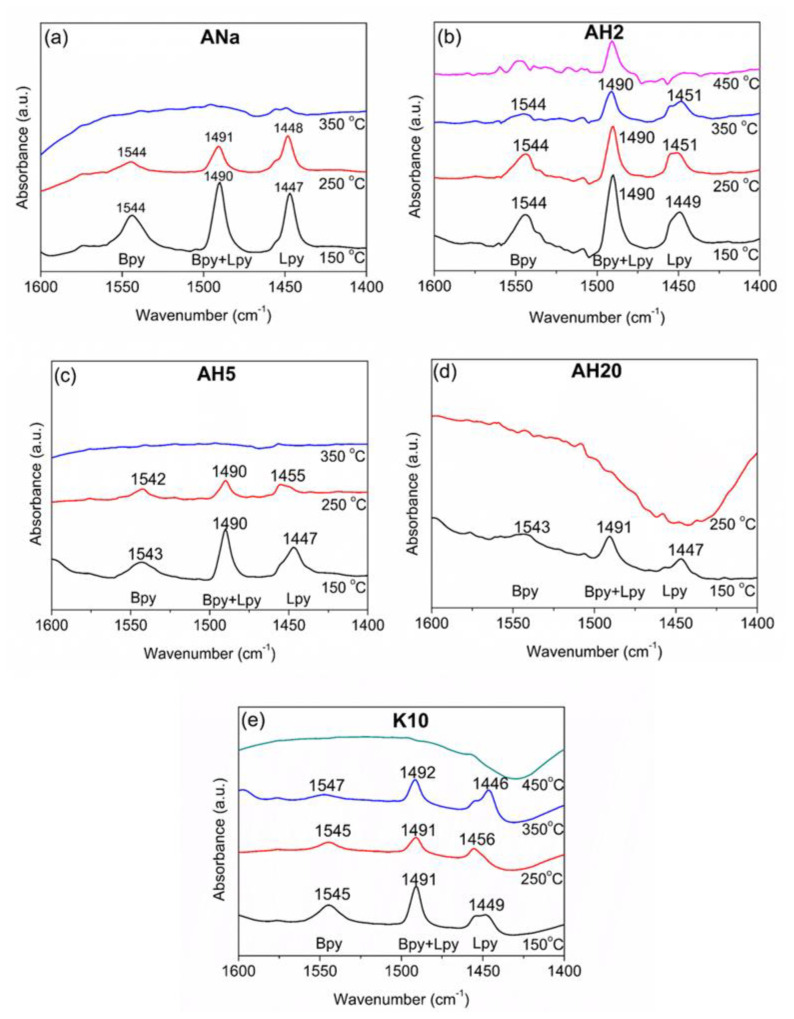
Fourier transform infrared (FTIR) spectra for pyridine after adsorption at 150 °C and desorption at increasing temperatures for clay minerals: (**a**) ANa; (**b**) AH2; (**c**) AH5; (**d**) AH20; (**e**) K10.

Figure 8 shows the FTIR-Pyr adsorption spectra for the clay minerals ANa, AH2, AH5, AH20 and K10 at 150 °C and the desorption spectra at 250, 350 and 450 °C, between 1600 and 1400 cm^−1^.

For samples ANa and AH5, no characteristic bands were observed at 350 °C, while for AH20, even at 250 °C it was not possible to detect them, thus no further spectra were recorded for those samples at higher temperatures. From now on, the acidic sites present only at 150 °C will be referred to as weak sites, those remaining after desorption at 250 °C as moderate strength sites and those remaining after desorption at 350 °C, as strong sites. No bands corresponding to Bpy or Lpy sites were observed at 450 °C within the detection limit of the equipment.

The absorption spectrum at 150 °C of the raw Mt ANa shows the presence of both Bpy sites (bands at 1544 cm^−1^) and Lpy sites (band at 1447 cm^−1^), besides an intense band at around 1490 cm^−1^ (Bpy + Lpy + HPy sites). These values are in the spectral range observed for FTIR-Pyr adsorption in different types of Mt [21,22,37,40]. After desorption at 250 °C, the intensities of the three bands decreased, revealing the presence of moderate strength Bpy and LPy sites; at 350 °C no bands were detected, indicating the absence of strong sites.

ANa corresponds to the raw Mt and AH2 results from its 2 h acidic activation with 4 mol L^−1^ H_2_SO_4_ solution. Although the AH2 spectrum after adsorption at 150 °C resembles that of its parent clay mineral, it is possible to observe that the acidic treatment increased the strength of both Bpy and Lpy sites, producing strong sites, as their respective bands were observed until 350 °C. Tyagi et al. [22] studied the surface acidity of Mt after being submitted to conventional hydrothermal, ultrasonic- and microwave-assisted acid digestion techniques by varying the time, the temperature and the H_2_SO_4_ concentration. According to them, the Lewis acidic sites are formed when the cations, Al^3+^, Mg^2+^ and Fe^3+^, usually present on the clay mineral edges, are exposed. Moreover, during the acidic activation, two phenomena occur in parallel: the exchange of the interlayer cations by H^+^ ions and the leaching of the octahedral cations Al^3+^, Mg^2+^ and Fe^3+^, with their consequent migration to the interlayer space, where they can dissociate water molecules, producing protons, which will act as Brønsted acidic sites.

As already depicted in the XRD diffractograms (Figure 2), there is a gradual loss of the Mt as the time for the acidic activation increases, and this structure breaking is possibly contributing to expose the cations that were originally coordinated within the octahedral sheet. In addition, according to the XRF results (Table 3), the leaching of those cations does occur, reinforcing the observations of Tyagi et al. [22] and explaining the changes in the FTIR-Pyr spectrum for AH2. However, as observed by Kooli and Jones [41] when studying the catalytic properties of a saponite subjected to different acidic treatments, the structure of the clay mineral is gradually destroyed as acidic activation proceeds, causing a reduction of the clay mineral layer charge and consequently the loss of its ability to retain the acidic protons. This can explain the lower intensity bands observed for AH5 at 150 and 250 °C, when compared to ANa and AH2, as well as the loss of the strong sites present in AH2. AH20, which was subjected to an even more severe acid treatment, for 20 h, presents very low intensity bands after adsorption at 150 °C and the absence of any acidic site after desorption at 250 °C. The commercial clay mineral K10 presents weak, moderate strength and strong sites, and it is noticeable that the Lpy bands are remarkably more intense than the Bpy ones for all desorption temperatures.

In order to calculate the concentration of acidic sites per mass unit of the sample, the molar extinction coefficients of the Mt-Lpy and Mt-Bpy bands must be known. Many works [30,31] report these values for zeolites and other solid acid catalysts, but not for Mt. Therefore, the quantification of Bpy and Lpy sites was made through the ratio of the integrated areas for each band area corresponding to Brønsted and Lewis acidity, according to Equations (1) and (2), where $A_{\mathrm{Bpy},\;T}$ corresponds to the integrated area of the band around 1545 cm^−1^ and $A_{\mathrm{Lpy},\;T}$ to the integrated area of the band around 1455 cm^−1^.

(1) $$\%\;\mathrm{Bpy}\;\mathrm{sites}=\frac{A_{\mathrm{Bpy},\;T}}{A_{\mathrm{Bpy},\;T}+A_{\mathrm{Lpy},\;T}}$$

(2) $$\%\;\mathrm{Lpy}\;\mathrm{sites}=\frac{A_{\mathrm{Lpy},\;T}}{A_{\mathrm{Bpy},\;T}+A_{\mathrm{Lpy},\;T}\;}$$

**Figure 9:**
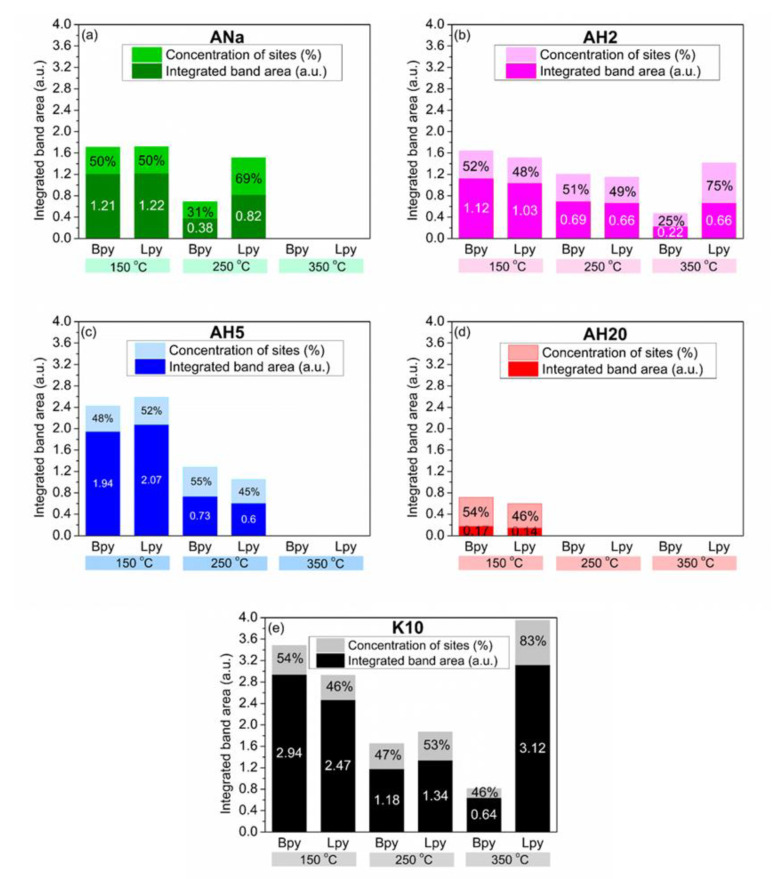
Calculated ratios for Bpy and Lpy acidic sites after pyridine adsorption at 150 °C and desorption at 250 and 350 °C: (**a**) ANa; (**b**) AH2; (**c**) AH5; (**d**) AH20; (**e**) K10.

The results of the calculations, reported in Figure 9, provide important insights to understand the better or worse performance of the samples in regard to the results of the LOI flammability test (Table 6). Firstly, comparing ANa (Figure 9a) and AH5 (Figure 9c), whose intumescent composites present LOI values of 35% and 38%, respectively, it is possible to observe that both clay minerals have weak and moderate strength acidic sites, but that the latter are distributed differently at 250 °C. ANa presents a large excess (more than twice) of Lpy sites (69%) in comparison to its Bpy sites (31%). In contrast, AH5 shows a slight excess of Bpy sites (55%) relative to Lpy (45%), as well as an enhancement of 3 LOI units. Also, the AH2 intumescent composites show a 35% LOI, but a different line of reasoning is needed to compare this result with that for AH5. By comparing Figure 9b,c, one can say that both AH2 and AH5 present similar distribution of LPy and Bpy sites at 250 °C, but the presence of strong sites in AH2 (at 350 °C) and their absence in AH5 are the main factors responsible for the difference in the LOI performance. Thus, the presence of strong sites seems to impair the flame-retardancy properties.

A comparison between the performance of the AH2 and ANa composites, both presenting a LOI value of 35%, provides important information concerning the role played by the strength and the nature of the acidic sites. AH2 presents the advantage of possessing Bpy and Lpy moderate strength sites distributed almost in the same proportion, with no excess of Lpy relative to Bpy. However, the presence of strong acidic sites appears to be a disadvantage. In turn, the ANa clay mineral does not contain strong acidic sites, which is a positive point, but presents a high excess of Lewis acidic sites relative to Brønsted ones at 250 °C, which counts negatively. Thus, there is a competition between the nature (Lewis or Brønsted) and the strength (moderate strength or strong) of the sites. In the case of ANa and AH2, the advantages and disadvantages of one compensate the advantages and disadvantages of the other, culminating in the same result for the LOI test.

Furthermore, AH2 and K10 (Figure 9e), exhibit a similar distribution of both types of site at 250 °C, but now the crucial difference resides in the distribution of the strong sites. K10 has 17% of Bpy and 83% of Lpy sites, whereas AH2 has 25% of Bpy sites and 75% of Lpy sites. The higher ratio (Bpy/Lpy = 1/3) in AH2 compared to almost 1/5 in K10, confers to its intumescent composites a better performance in the LOI test (35% compared to 33% for K10).

The importance of the medium strength Brønsted acidity is confirmed when examining the results obtained for AH20 (Figure 9d), whose intumescent composites achieved 27% LOI. The absence of any acidic sites at 250 °C, plus the loss of the lamellar structure can impair the action of AH20 as a catalyst of the esterification reaction. Besides, the system PP/IF/AH20 showed an even worse performance than the system without any clay mineral, PP/IF (30% LOI). A possible explanation is that the destroyed lamellas of AH20 give rise to a physical barrier between the reactants, minimizing the probability of the esterification reaction and, consequently, weakening the flame-retarding properties. This negative effect was also remarked on by Tang et al. [24], who justified poorer flame retarding properties by an ablative reassembling of the silicate layers, which can hinder NH_3_, the blowing agent, impairing the material to swell. The swelling process is important in order to increase the barrier distance between the outside char layer exposed to the flames and the inner polymeric matrix, slowing down the transference of heat and, therefore, the composite degradation kinetics. Many authors investigated the influence of the acidity of Mt on the catalysis of organic reactions [21,22,37,39,40,42]. Jha et al. [21] prepared metal cation-exchanged Mt catalysts and observed the highest conversion of p-cresol to 2,2-methylenebis (4-methyl phenol) with the catalyst exhibiting the highest Brønsted acidity. Reddy et al. [37] studied the role of acidic sites present in Al^3+^ exchanged raw and K10 Mt, aluminium pillared Mt catalysts and Mt treated with HCl solution. They observed a good correlation between the Brønsted acidic sites concentration and the catalytic activity in the esterification of propionic acid with p-cresol and that Lewis acidic sites, even in high concentration, do not catalyse the reaction. Based on these well-established literature results, it is quite reasonable to consider that Mt catalyses the reaction between APP and PER for the systems investigated in this study, at temperatures below 280 °C, to produce the phosphate esters, which gives rise to the precursors of char. This can be the reason why the presence of Brønsted acidic sites (i.e., moderate strength Bpy sites) at temperatures below 280 °C, is imperative, as they act as a proton-donors for that reaction to occur. Consequently, the char could be formed earlier, enhancing the protection of the inner polymeric matrix, and achieving a better flame-retarding property. Moreover, the presence of strong sites, especially Lewis ones, seems to impair the flame retardancy. One possible explanation is that they can act as traps for the ammonia released in the gas phase from the APP decomposition. Once they retain some of the ammonia molecules, the protective layer partially loses its ability to swell, thus facilitating the transfer of energy between the outer and the inner environments, which is not desirable for an intumescent system. That is why the PP/IF/AH5 system, whose clay mineral contains only weak and moderate strength sites, with excess of Bpy relative to Lpy, showed the best performance in the LOI test.

## 4. Conclusions

The acidic activation of Mt has proven to be an efficient procedure to change the concentration of acidic sites (as shown by the TPD-NH_3_ results), as well as their nature and strength, as proven by FTIR-Pyr adsorption. The XRF data showed that the exchange of Na^+^ from the clay mineral interlamellar space by the H^+^ from the acid was successful, enabling the creation of Brønsted acidic sites. Furthermore, the longer the time it took for acidic activation the greater was the lixiviation of the octahedral sheet cations, which corroborates the loss of Mt organization observed in XRD diffractograms. The acidic treatment also contributed to change the specific surface area and the pore volume of the clay minerals. The similarity of those parameters observed for ANa, AH2 and AH5 indicated that the Mt crystalline phase organization was preserved, whereas the very low specific surface area and high pore diameter obtained for AH20 confirm the loss of that organization. The particle size dispersion achieves its maximum for AH2 and gradually decreases, becoming more homogeneous with further acidic treatment. The SEM images for the samples with the intumescent formulation show that the surfaces of the carbonaceous layers are rugged and hilly, whereas in the samples without the intumescent formulation these layers are not formed. This difference in the physical aspect responds for the increase of the thermal stability and of the final residue at high temperatures for the composites containing the intumescent formulation. Moreover, the performance in the flammability tests seems to be independent of the particles’ distribution in the PP matrix. The TGA results show that the incorporation of clay minerals in the intumescent composites raises the intumescent layer´s thermal stability, leading to an increase of the final residue, a factor that is closely related to the improvement of the flame-retardant properties. The addition of Mt increases the char degradation temperature in the systems with the intumescent formulation, no matter the nature and the strength of the acidic sites. All the intumescent composites, with and without the addition of clay mineral, achieved the best classification in the UL-94 test, V0, while the samples without IF could not be classified. The sole addition of the clay minerals to the polymeric matrix did not change significantly the LOI value for neat PP (17%). However, the opposite effect was observed when the IF was also added. The system PP/IF achieved 30% O_2_, whereas this value raised to 33 (for K10), 35 (for ANa and AH2) and 38% O2 (for AH5), once more showing a synergistic action between the clay minerals and the intumescent formulation. However, for the AH20 system this value was lower (27% O2) than that obtained for the PP/IF/composite. Finally, FTIR-Pyr adsorption results show that there is a competition between the nature and the strength of the acidic sites, which directly influences the flame-retardant properties. A better performance is achieved with an excess of Brønsted sites relative to the Lewis ones, whereas moderate-strength sites are preferable to the strong ones, as the latter tend to impair the flame-retarding properties. It is possible that the swelling process, which is fundamental for decreasing the degradation rate of the inner polymeric matrix, is weakened by the strong acid sites, as they can work as ammonia traps. Furthermore, flammability and FTIR-Pyr adsorption results point towards the necessity of the existence of these Brønsted acidic sites at a key temperature—until 280 °C for the APP-PER system studied in this work—as they take part in the esterification reaction between APP and PER. In summary, once the influence of both the strength and the nature of the acidic sites of Mt on the flame retardancy in intumescent composites is better understood, new catalysts can be developed containing the desired properties in order to develop materials with better flame-retarding performance.

## Figures and Tables

**Table 1:** Concentration (% *m*/*m*) of the components in each system studied and equations used to construct the theoretical thermogravimetric analysis (TGA) curves.

System	Polypropylene (PP)	Intumescent Formulation (IF)	Clay	*M*_theo_ (*T*)
PP/IF	70%	30%	-	*M*_theo_ (*T*) = 0.70 *M*_PP_ (*T*) + 0.30 *M*_IF_ (*T*)
PP/IF/clay	67%	30%	3%	*M*_theo_ (*T*) = 0.67 *M*_PP_ (*T*) + 0.30 *M*_IF_ (*T*) + 0.03 *M*_clay_ (*T*)

*M*_theo_ (*T*): theoretical mass loss. *M* (*T*): experimental mass loss.

**Table 2:** Criteria for Underwriters Laboratory UL-94 classification [28].

Criteria	V-0	V-1	V-2
Individual values of t_1_ and t_2_ for the 5 specimens	<10 s	<30 s	<30 s
(t1+t2) for the 5 specimens	<50 s	<250 s	<250 s
t3 values	<30 s	<60 s	<60 s
Combustion up to holding clamp (specimens completely burned)	No	No	No
Dripping of burning specimens (ignition of cotton batting)	No	No	Yes

**Table 3:** Chemical composition of the Mt. The ratios are presented in % m/m.

	MgO	Al_2_O_3_	SiO_2_	SO_3_	K_2_O	TiO_2_	Fe_2_O_3_	CaO	Cl	Na_2_O	*LDC
**ANa**	2.00	12.8	62.7	<0.1	0.13	0.63	5.1	0.57	0.15	0.48	15.4
**AH2**	0.96	10.7	72.0	<0.1	0.12	0.70	3.0	<0.1	<0.1	<0.1	12.5
**AH5**	0.78	8.9	75.5	0.12	0.12	0.71	2.1	<0.1	<0.1	<0.1	11.7
**AH20**	<0.1	1.4	90.8	0.31	0.12	0.75	0.3	<0.1	<0.1	<0.1	6.2
**K10**	1.80	15.6	60.3	0.12	1.20	0.47	3.7	0.26	<0.1	0.30	16.3

*LDC = lost due to calcination.

**Table 4:** Textural properties of the clay mineral samples.

Sample	Specific Surface Area (m^2^ g^−1^)	Pore Volume (cm^3^ g^−^^1^)	Desorption Average Pore Diameter (Å)
**ANa**	104	0.16	53
**AH2**	145	0.18	51
**AH5**	137	0.18	53
**AH20**	87	0.23	139
**K10**	178	0.22	51

**Table 5:** Experimental and theoretical data for TGA and dTG curves for the PP/FI and PP/FI/clay mineral systems.

Sample	Experimental Data				Theoretical Data			
	% Mass at 900 °C	T_onset_ (°C)	^a^ T_peak1_ (°C)	^a^ T_peak2_ (°C)	% Mass at 900 °C	T_onset_ (°C)	T_peak1_ (°C)	T_peak2_ (°C)
**PP/IF**	2.0	275	378/394	642	1.8	290	414	680
**PP/IF/Ana**	9.1	271	370	697	4.3	311	414	680
**PP/IF/AH2**	12.5	273	374	682	4.4	311	414	680
**PP/IF/AH5**	11.4	290	399	642	4.4	311	414	680
**PP/IF/AH20**	12.2	293	394	642	4.6	311	414	680
**PP/IF/K10**	10.2	288	369	664	4.3	311	414	680

^a^ T_peak1_ and T_peak2_: first and second most intense dTG peaks, respectively.

**Table 6:** Limit oxygen index (LOI) results for samples with and without intumescent formulation.

Sample Without IF	LOI ± 1 (% O_2_)	Sample With IF	LOI ± 1 (% O_2_)
PP	17	PP/IF	30
PP/ANa	18	PP/IF/Ana	35
PP/AH2	18	PP/IF/AH2	35
PP/AH5	18	PP/IF/AH5	38
PP/AH20	18	PP/IF/AH20	27
PP/K10	18	PP/IF/K10	33
